# Revision of fossil Metretopodidae (Insecta, Ephemeroptera) in Baltic amber – Part 4: Description of two new species of *Siphloplecton* Clemens, 1915, with notes on the new *S. jaegeri* species group and with key to fossil male adults of *Siphloplecton*

**DOI:** 10.3897/zookeys.898.47118

**Published:** 2019-12-10

**Authors:** Roman J. Godunko, Christian Neumann, Arnold H. Staniczek

**Affiliations:** 1 Biology Centre of the Czech Academy of Sciences, Institute of Entomology, Branišovská 31, 37005 České Budějovice, Czech Republic Biology Centre of the Czech Academy of Sciences České Budějovice Czech Republic; 2 Department of Invertebrate Zoology and Hydrobiology, University of Łódź, Banacha 12/16, 90237, Łódź, Poland University of Łódź Łódź Poland; 3 Museum für Naturkunde, Leibniz Institute for Evolution and Biodiversity Science, 10115, Berlin, Germany Museum für Naturkunde Berlin Germany; 4 Department of Entomology, Stuttgart State Museum of Natural History, Rosenstein 1, 70191, Stuttgart, Germany Stuttgart State Museum of Natural History Stuttgart Germany

**Keywords:** Fossil insects, mayflies, Siphlonuroidea, new species, species group, Eocene

## Abstract

The *Siphloplecton jaegeri* species group is established here for three extinct species, namely for the earlier described *Siphloplecton jaegeri* Demoulin, 1968, and for two new species from Eocene Baltic amber, *Siphloplecton landolti***sp. nov.** and *Siphloplecton studemannae***sp. nov.** Based on the well-preserved specimens of these species, a diagnosis is provided for the newly established species group. Representatives of the *S. jaegeri* species group are characterized by the presence of large, medially contiguous eyes, stout pointed setae along the outer margin of the foretibia, three intercalaries in the cubital field of the forewing, and elongated penis lobes, which are apically triangular or rounded, medially contiguous, and with a V-shaped cleft apically. Further new specimens of the *S. jaegeri* species group are documented that cannot be attributed to species level due to their poor preservation. Finally, a key to male adults of fossil species of *Siphloplecton* is given.

## Introduction

Metretopodidae Traver, 1935 is a small, monophyletic mayfly family of Holarctic distribution, comprising 11 extant species in three genera (Berner 1978; Kluge 2004). While *Metretopus* Eaton, 1901 is distributed throughout the Holarctic, the extant distribution of *Siphloplecton* Clemens, 1915 is restricted to the Nearctic, and *Metreplecton* Kluge, 1996 is only found in the Palearctic. Recently, Staniczek and Godunko (2012, 2015, 2016) provided an overview of the taxonomic history of fossil Metretopodidae, which are only known from Eocene Baltic amber. They addressed the two fossil species of *Siphloplecton* Clemens, 1915 treated by Demoulin (1968): *S. jaegeri* Demoulin, 1968 was redescribed, whereas the lectotype of *S. macrops* (Pictet-Baraban & Hagen, 1856) was considered to be lost (Staniczek and Godunko 2012). Additionally, *S. barabani* Staniczek & Godunko, 2012; *S. picteti* Staniczek & Godunko, 2012; *S. hageni* Staniczek & Godunko, 2012; and *S. demoulini* Staniczek & Godunko, 2012 were described from historical material (Staniczek and Godunko 2012). The hitherto described fossil material of *Metretopus* (Demoulin, 1970) was also revised (Staniczek and Godunko 2015), and *M. dividus* Staniczek & Godunko, 2015 was described from new material. In a third part of the revision of fossil Metretopodidae, the rediscovery of the *S. macrops* lectotype was documented (Staniczek and Godunko 2016). Based on new material, complementary descriptions of *S. picteti* and *S. barabani* were given, and the *picteti* and *demoulini* species groups were established within *Siphloplecton*. Finally, two new species, *S. sartorii* Staniczek & Godunko, 2016 and *S. gattolliati* Staniczek & Godunko, 2016, were described from newly emerged material and attributed to these species groups, respectively (Staniczek and Godunko 2016).

In this fourth contribution to the knowledge of fossil Metretopodidae, we establish the *jaegeri* species group within *Siphloplecton* based on newly available material. Two recently discovered, well preserved male imagines from Baltic amber provide new data for a complementary description of the fossil species *Siphloplecton jaegeri*; two new species, *S. landolti* sp. nov. and *S. studemannae* sp. nov., are described and attributed to this species group; and finally, further two new specimens of the *S. jaegeri* group are documented that cannot be attributed to species level due to their poor conservation.

## Material and methods

All specimens examined in this study are housed in the following collections (for details see under *Material examined* in the species descriptions):

**CCHH** collection of Christel and Hans Werner Hoffeins, Hamburg, Germany (later to be housed at Senckenberg Deutsches Entomologisches Institut, Müncheberg, Germany (SDEI));

**MNB**Museum für Naturkunde, Berlin, Germany;

**MNHK**Museum für Naturkunde, Berlin, Germany, Institute of Systematics and Evolution of Animals, PAS, Kraków, Poland;

**MNHN**Muséum national d’Histoire naturelle, Paris, France;

**SMNS**Staatliches Museum für Naturkunde Stuttgart, Germany.

Drawings were made with a camera lucida on an Olympus SZX7, a Leica S8 APO and a Leica M205 C stereo microscopes. Multiple photographs with different depth of field were taken through a Leica Z16 APO Macroscope using Leica Application Suite v. 3.1.8. Photo stacks were processed with Helicon Focus Pro 6.4.1 to obtain combined photographs with extended depth of field, and subsequently enhanced with Adobe Photoshop CS3.

## Systematic palaeontology

### Order Ephemeroptera Hyatt & Arms, 1890

#### Family Metretopodidae Traver, 1935

**Genus *Siphloplecton* Clemens, 1915**

##### Siphloplecton jaegeri

Taxon classificationAnimaliaEphemeropteraMetretopodidae

species group

BD1E6FA5-39BE-55EB-A28C-876186AD97D3

###### Diagnosis.

Male imago: (1) eyes large, medially contiguous; (2) outer margin of foretibia with several stout, pointed setae; (3) cubital field with three long intercalary veins: one long intercalary vein connected with CuA by one or several crossveins; one pair of intercalary veins situated close to CuP and at least connected with CuA (or additionally with CuP) by a crossvein; (4) plate angulate, mediocaudally only shallowly incised; incisions with relatively small pronounced triangular projections; with or without medial projection, if present, small and bluntly pointed apically; (5) forceps relatively long; basal segment conical, without apical hump on inner margin; (6) penis stem elongated; (7) penis significantly surmounting distal end of plate, reaching 0.25−0.30× length of forceps segment 2; (8) penis lobes elongated, apically triangular or rounded, medially contiguous, with V-shaped cleft apically.

###### Species composition.

*Siphloplecton jaegeri* Demoulin, 1968, *Siphloplecton landolti* sp. nov., *Siphloplecton studemannae* sp. nov., *Siphloplecton* sp. 5, *Siphloplecton* sp. 6.

##### Siphloplecton jaegeri

Taxon classificationAnimaliaEphemeropteraMetretopodidae

Demoulin, 1968

7FD9035A-A5F6-5731-941A-ACB9B93F19C4

**Figure 1.**
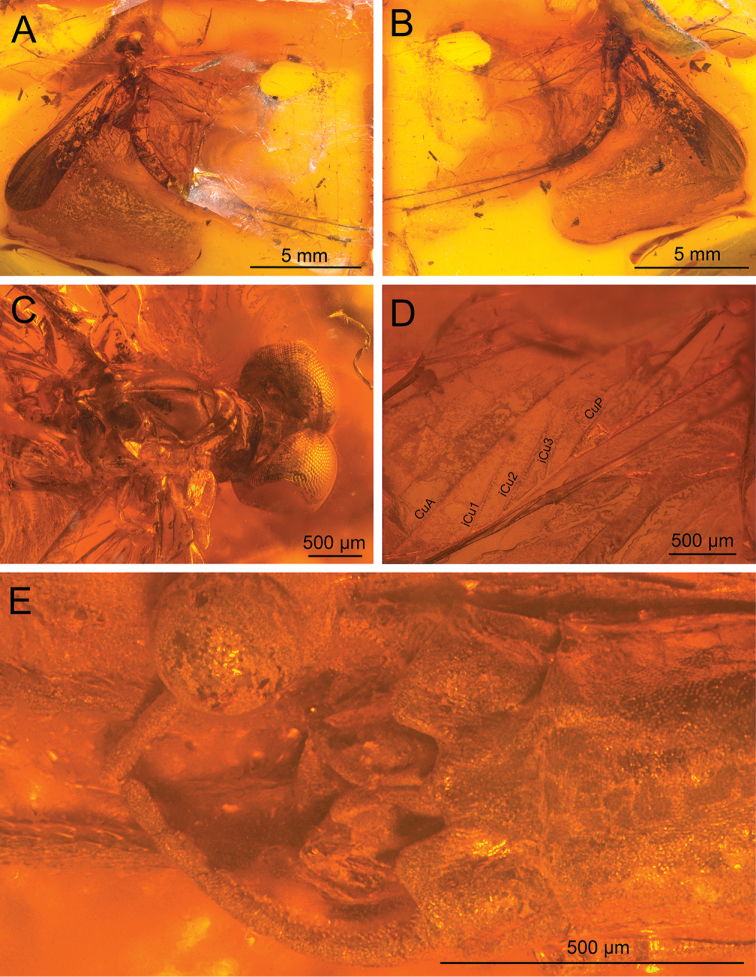
*Siphloplecton jaegeri* Demoulin, 1968, MNB, MB.I 7370, male imago (photographs) **A** general dorsal view **B** general ventral view **C** head and thorax in dorsal view **D** right forewings (details of cubital field in ventral view) **E** genitalia in ventral view.

**Figure 2.**
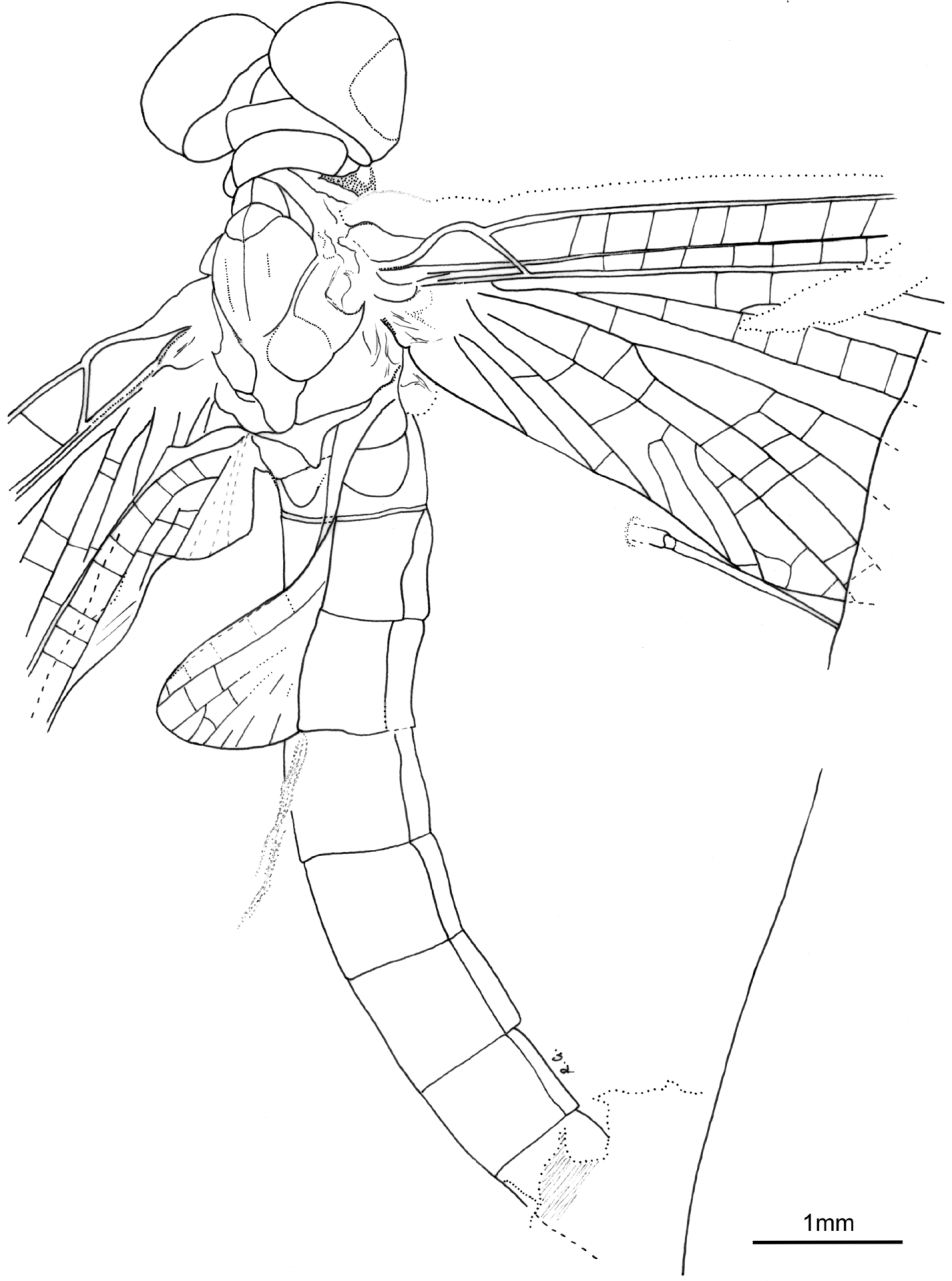
*Siphloplecton jaegeri* Demoulin, 1968, MNB, MB.I 7370, male imago (line drawing): general dorsal view.

**Figure 3.**
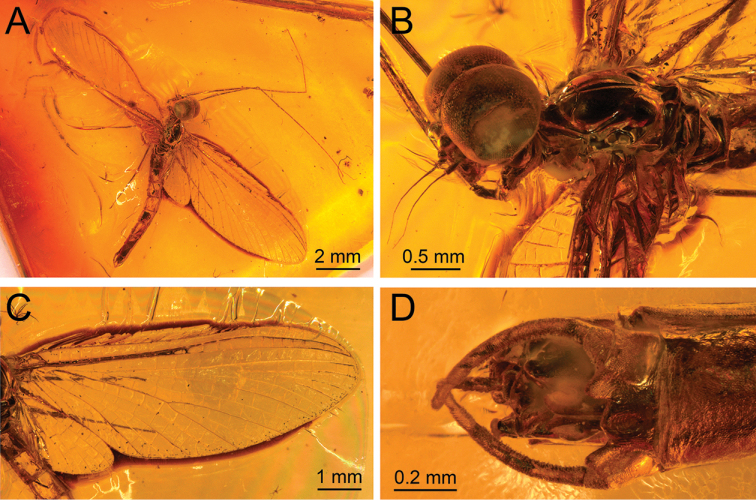
*Siphloplecton jaegeri* Demoulin, 1968, MNHN, 4655 BA, male imago (photographs) **A** general dorsal view (tip of abdomen twisted to ventral side) **B** head and thorax in dorsolateral view **C** right fore- and hind wing in dorsal view **D** genitalia in ventral view.

**Figure 4.**
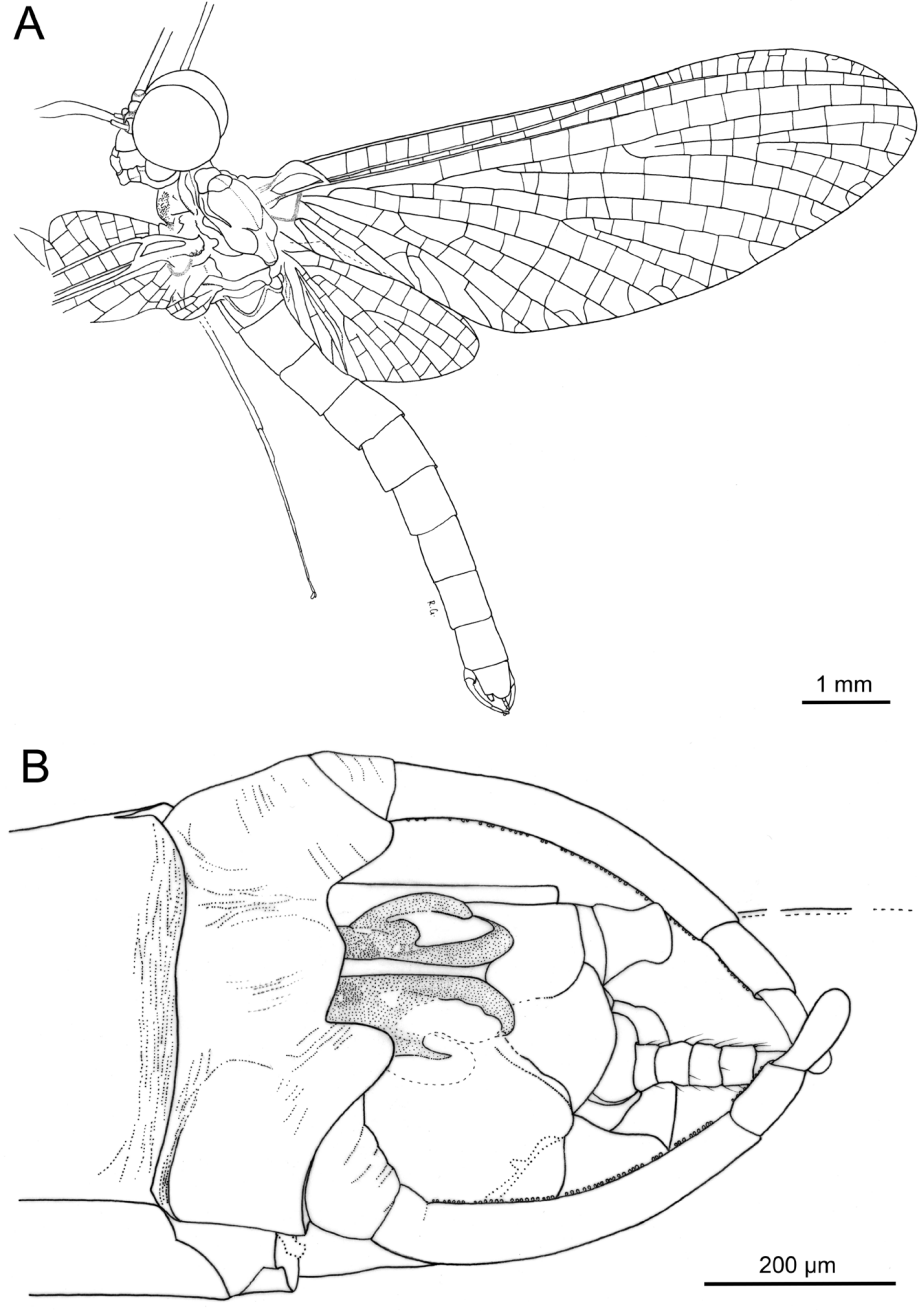
*Siphloplecton jaegeri* Demoulin, 1968, MNHN, 4655 BA, male imago (line drawings) **A** general dorsal view **B** genitalia in ventral view.

Figures 1 , 2 , 3 , 4 ; Table 1

Siphloplecton jaegeri Demoulin, 1968 – *Deutsche Entomologische Zeitschrift*: 252, figs 18a, c (description, designation of holotype)
Siphloplecton jaegeri Demoulin, 1968 – Staniczek and Godunko 2012, Paleodiversity: 73, figs 10a, b, 11a−c (redescription of holotype). For complete list of synonymies see Staniczek and Godunko (2012: 73).

###### Material examined.

Male imago in Baltic amber (Eocene), MNB, MB.I 7370, specimen labelled as: “6. Pseudoneuroptera III Ephemeridae”; “Museum für Naturkunde Berlin”; “Paläontologisches Museum”; “Slg.: Künow Inv. Nr.: Nr. 268–294 nur noch 9 Stück vorgefunden”; “Ephemeriden”; “Siphloplecton cf. jaegeri ♂ imago Nr.: 271” (Figs 1A–E, 2).

Well preserved specimen, visible in dorsoventral aspect. Wings completely preserved (Fig. 1A, B); posterior margin of left forewing and hind wings twisted. Ventral side of head and prosternum not visible, view obstructed by resin influx and cracks in stone. Foremargin and distal part of left forewing and entire left hind wing dirty brownish coloured; several dark spots on remaining part of left forewing. Such irregular pigmentation is a side effect of the specific conditions of fossilization, and must not be confused with the natural pigmentation of *Siphloplecton* wings (the right wings of the same specimen are colourless and translucent). Right fore- and left middle legs lost. Cerci partly damaged.

For measurements see Table 1.

**Table 1.** Measurements of fossil representatives of the *Siphloplecton jaegeri* species group.

Adult characters	*Siphloplecton jaegeri* [MNB, MB.I 7370, male imago] (mm)	*Siphloplecton jaegeri* [MNHN, 4655 BA, male imago] (mm)	*Siphloplecton landolti* sp. nov. [SMNS, BB–2377, holotype, male imago] (mm)	*Siphloplecton studemannae* sp. nov. [SMNS, BB–2626, holotype, female imago] (mm)	*Siphloplecton studemannae* sp. nov. [MNHK, MP/1626, paratype, female imago] (mm)	*Siphloplecton* sp. 5 [CCHH, BaB 1159/5, male imago] (mm)	*Siphloplecton* sp. 6 [MNB, MB.I 7372, male subimago] mm)
Length of body	9.00	9.08	10.88	10.04	9.48	9.50	8.50
Length of right foreleg	–	9.64	–	–	–	–	5.11
Length of femur	–	1.80	–	–	–	–	1.77
Length of tibia	–	2.00	–	–	–	–	1.05
Length of tarsus	–	5.84	–	–	–	–	2.29
Tarsomere 1	–	1.40	–	–	–	–	0.48
Tarsomere 2	–	1.40	–	–	–	–	0.45
Tarsomere 3	–	1.44	–	–	–	–	0.48
Tarsomere 4	–	1.20	–	–	–	–	0.53
Tarsomere 5	–	0.40	–	–	–	–	0.35
Length of left foreleg	8.54	9.40	3,44*	4.04*	–	13.44	5.04
Length of femur	1.75	1.80	1.04*	2.02*	–	3.12	1.73
Length of tibia	1.95	1.88	2.40	1.52	–	2.72	1.10
Length of tarsus	4.84	5.72	4.64*	0.50*	–	7.60	2.21
Tarsomere 1	1.13	1.32	1.80	0.50	–	2.08	0.45
Tarsomere 2	1.13	1.32	2.00	–	–	1.88	0.45
Tarsomere 3	1.05	1.48	0.84*	–	–	1.64	0.50
Tarsomere 4	1.15	1.20	–	–	–	1.44	0.48
Tarsomere 5	0.38	0.40	–	–	–	0.56	0.33
Length of right middle leg	–	3.52	–	4.28	–	7.24	3.86
Length of femur	–	1.32	–	1.52	–	2.88	1.58
Length of tibia	–	0.40	–	1.20	–	1.60	0.85
Length of tarsus	–	1.80	–	1.56	–	2.76	1.43
Tarsomere 1	–	0.40	–	0.60	–	0.88	0.30
Tarsomere 2	–	0.44	–	0.32	–	0.68	0.40
Tarsomere 3	–	0.40	–	0.24	–	0.52	0.30
Tarsomere 4	–	0.40	–	0.22	–	0.32	0.23
Tarsomere 5	–	0.16	–	0.18	–	0.36	0.20
Length of left middle leg	2.11*	3.52	3.48*	4.31	4.09	7.28	3.84
Length of femur	–	1.32	1,80	1.52	1.63	2.92	1.55
Length of tibia	0.63*	0.40	1.04	1.21	1.00	1.64	0.85
Length of tarsus	1.48	1.80	0.64*	1.58	1.46	2.72	1.44
Tarsomere 1	0.35	0.40	0.64*	0.62	0.44	0.80	0.30
Tarsomere 2	0.50	0.40	–	0.34	0.45	0.72	0.40
Tarsomere 3	0.25	0.40	–	0.22	0.13	0.48	0.33
Tarsomere 4	0.20	0.40	–	0.20	0.21	0.36	0.20
Tarsomere 5	0.18	0.20	–	0.20	0.23	0.36	0.20
Length of right hind leg	3.52	3.10	–	4.52	2.68*	6.84	3.54
Length of femur	1.36	1.32	–	1.68	1.45	2.48	1.63
Length of tibia	0.78	0.40	–	1.10	0.90	1.64	0.70
Length of tarsus	1.38	1.38	–	1.74	0.33*	2.72	1.21
Tarsomere 1	0.35	0.44	–	0.46	0.33	0.88	0.30
Tarsomere 2	0.33	0.36	–	0.44	–	0.72	0.30
Tarsomere 3	0.25	0.22	–	0.38	–	0.48	0.20
Tarsomere 4	0.20	0.16	–	0.24	–	0.36	0.18
Tarsomere 5	0.25	0.20	–	0.22	–	0.28	0.23
Length of left hind leg	3.43	3.43	3.40*	4.42	3.01*	6.84	3.65
Length of femur	1.30	1.32	1.92	1.72	1.48	2.48	1.65
Length of tibia	0.75	0.44	0.84	1.06	0.88	1.60	0.75
Length of tarsus	1.38	1.36	0.64*	1.64	0.65*	2.76	1.25
Tarsomere 1	0.35	0.40	0.64*	0.42	0.30	0.84	0.30
Tarsomere 2	0.35	0.40	–	0.42	0.35*	0.76	0.35
Tarsomere 3	0.25	0.20	–	0.38	–	0.52	0.25
Tarsomere 4	0.18	0.16	–	0.22	–	0.32	0.15
Tarsomere 5	0.25	0.20	–	0.20	–	0.32	0.20
Length of right forewing	8.13	8.20	11.60	8.80	9.95	9.84	8.75
Length of left forewing	8.25	8.08*	11.80	8.60	6.88*	9.86	8.55
Length of right hind wing	3.15	2.70	3.60	–	3.25	3.50	2.75
Length of left hind wing	3.20	–	3.25*	2.92	3.30	3.52	2.80
Hind/forewings length ratio	0.39	0.39	0.31	0.34	0.33	0.36	0.32
Length of right cercus	4.25*	–	2.84*	2.24*	3.03	–	4.25*
Length of left cercus	4.20*	–	3.16*	2.32*	4.50	8.80	4.00*

* − preserved part; “−” – not preserved.

Male imago in Baltic amber (Eocene), MNHN, 4655 BA (Figs 3–4).

The specimen is visible in dorsoventral and, partly, lateral aspect. Head and thorax ventrally with “Verlumung”. Right fore- and hind wings fully preserved; left pair of wings partly twisted; details of cubital field not discernible. Cerci lost.

Colour yellow to yellowish-brown with darker thorax, but generally paler than all other known specimens of *S. jaegeri*. Wings hyaline, translucent, without any pigmentation.

For measurements see Table 1.

###### Description of specimens.

General colouration from pale (yellow to yellowish-brown), to dark brown (yellowish-brown to intensively brown); details of wing colouration are described above.

***Head*** uniformly brown. Eyes large, well developed, medially contiguous (Figs 1C, 2, 3A, 4A), slightly flattened laterally (MNB specimen); ocelli of MNHN specimen with slightly paler apical part and darker basally; antennae light brown, longer than head (Fig. 3A); ocelli and antennae of MNB specimen not visible due to resin influxes (Fig. 1A–C).

***Thorax*** brown with dirty maculation dorsally (MNB specimen), yellowish-brown with markedly darker sterna (MNHN specimen); mesonotal suture stretched backwards medially; lateroparapsidal suture elongated, without surrounding pigmentation; furcasternal protuberances of mesothorax fused.

***Wings*** hyaline. Pterostigma with at least 6 anastomosed veins. Cubital field of right forewing with well-developed intercalary vein (iCu1) close to CuA and basally directly connected to it, followed by one pair of intercalaries (iCu2, iCu3) basally connected to each other and connected to CuA (in MNHN specimen also to CuP) by a short crossvein (Figs 1D, 2, 4A). Hind wings with triads RS, MA and MP (Fig. 4A); preserved part of hind wings 0.39× forewing length (MNB specimen). Hind wings of MNHN specimen with triads RS, MA and MP, poorly visible distally; preserved part of hind wings 0.35× forewing length. Costal process small.

***Legs*** brownish; tibiae and tarsi darker than femora; structure and proportions of leg segments similar to those of holotype of *S. jaegeri*; outer margin of foretibia with pointed setae; measurements of leg segments in Table 1.

***Abdominal*** segments well preserved, paler than thorax. Shape of styliger and penis lobes (Figs 1E, 3D, 4B) similar to those of *S. jaegeri* holotype (Demoulin 1968: 252, figs 18a, c, Staniczek and Godunko 2012: 74, fig. 11c). Styliger plate angulate, deeply incised with three prominent projections; medial projection markedly broad; basal segment of forceps basally (a) markedly narrower than adjoining apical part of plate (b) (a/b = 0.57); forceps 4-segmented; segment 2 longest, segment 4 approximately 2.47 to 2.65 times as long as wide; length ratio of segment 3 to segment 4 approximately 0.9:1. Penis lobes elongated, medially incised, triangular, with relatively inconspicuous incision between lateral and medial penis sclerites. Surface details of penis lobes not visible.

***Paracercus*** vestigial, 5-segmented.

###### Comments.

Some minor differences regarding the proportions of the fore/hind wings and forceps segments between the holotype of *S. jaegeri* and the specimens described above are present. However, we attribute these specimens to *S. jaegeri* due to the presence of 1+2 intercalaries in the cubital field, pointed setae along outer margin of foretibia, and the shape of plate and penis lobes.

##### Siphloplecton landolti sp. nov.

Taxon classificationAnimaliaEphemeropteraMetretopodidae

AA629EF8-A7B5-5C92-8F18-0E561D8DBDAA

http://zoobank.org/F5338779-9D55-48B5-9542-7357704A8A64

**Figure 5.**
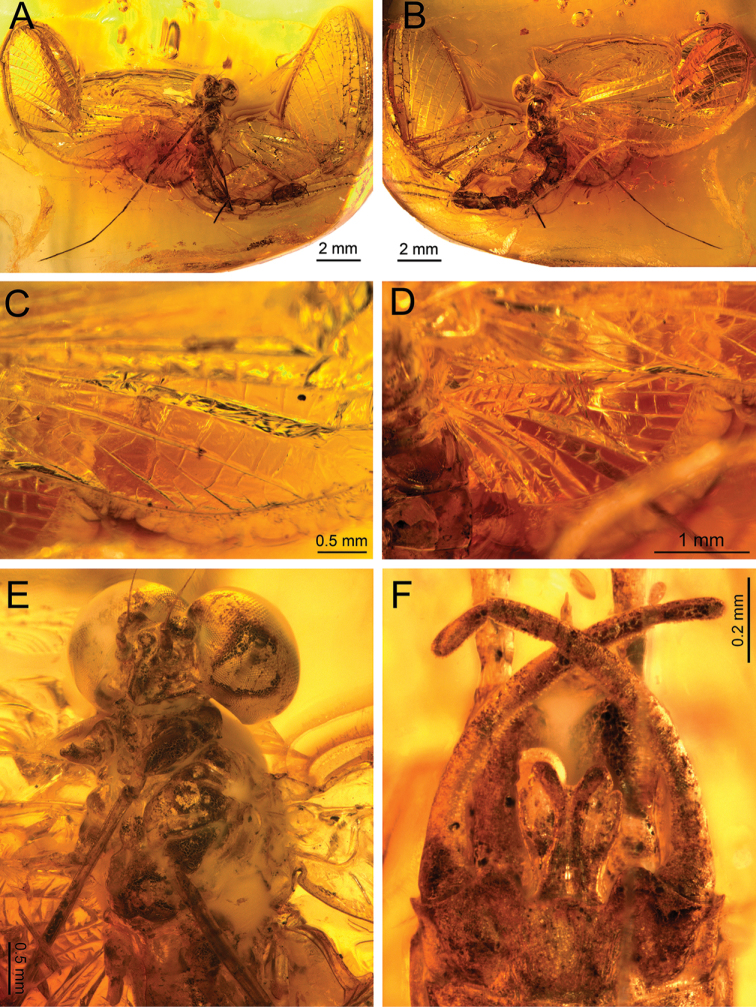
*Siphloplecton landolti* sp. nov., SMNS, BB–2377, holotype, male imago (photographs) **A** general dorsal view **B** general ventral view **C** cubital field of right forewing **D** right hind wing in dorsal view **E** head and thorax in ventral view **F** genitalia in ventral view.

**Figure 6.**
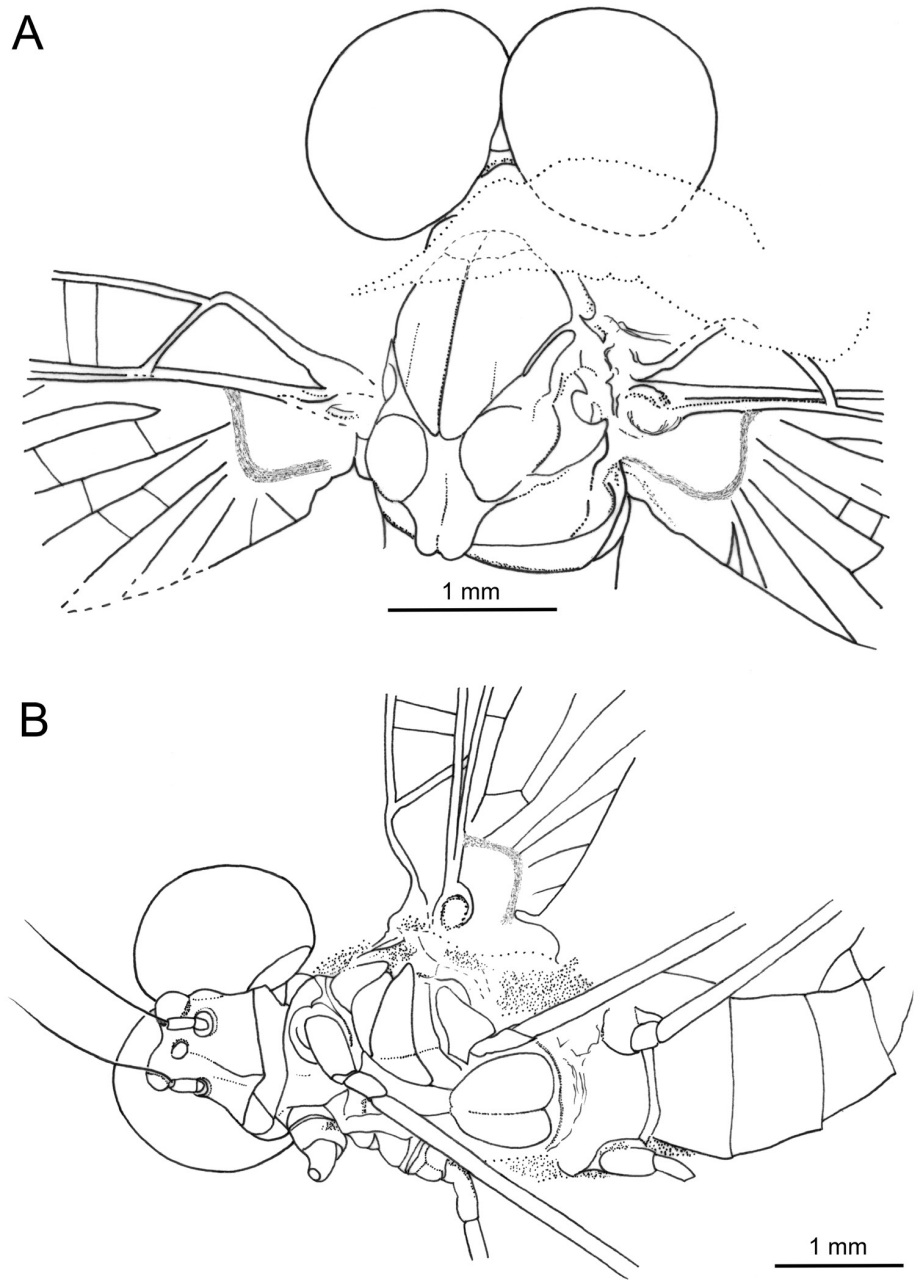
*Siphloplecton landolti* sp. nov., SMNS, BB–2377, holotype, male imago (line drawings) **A** head and thorax in dorsal view **B** head and thorax in ventral view.

**Figure 7.**
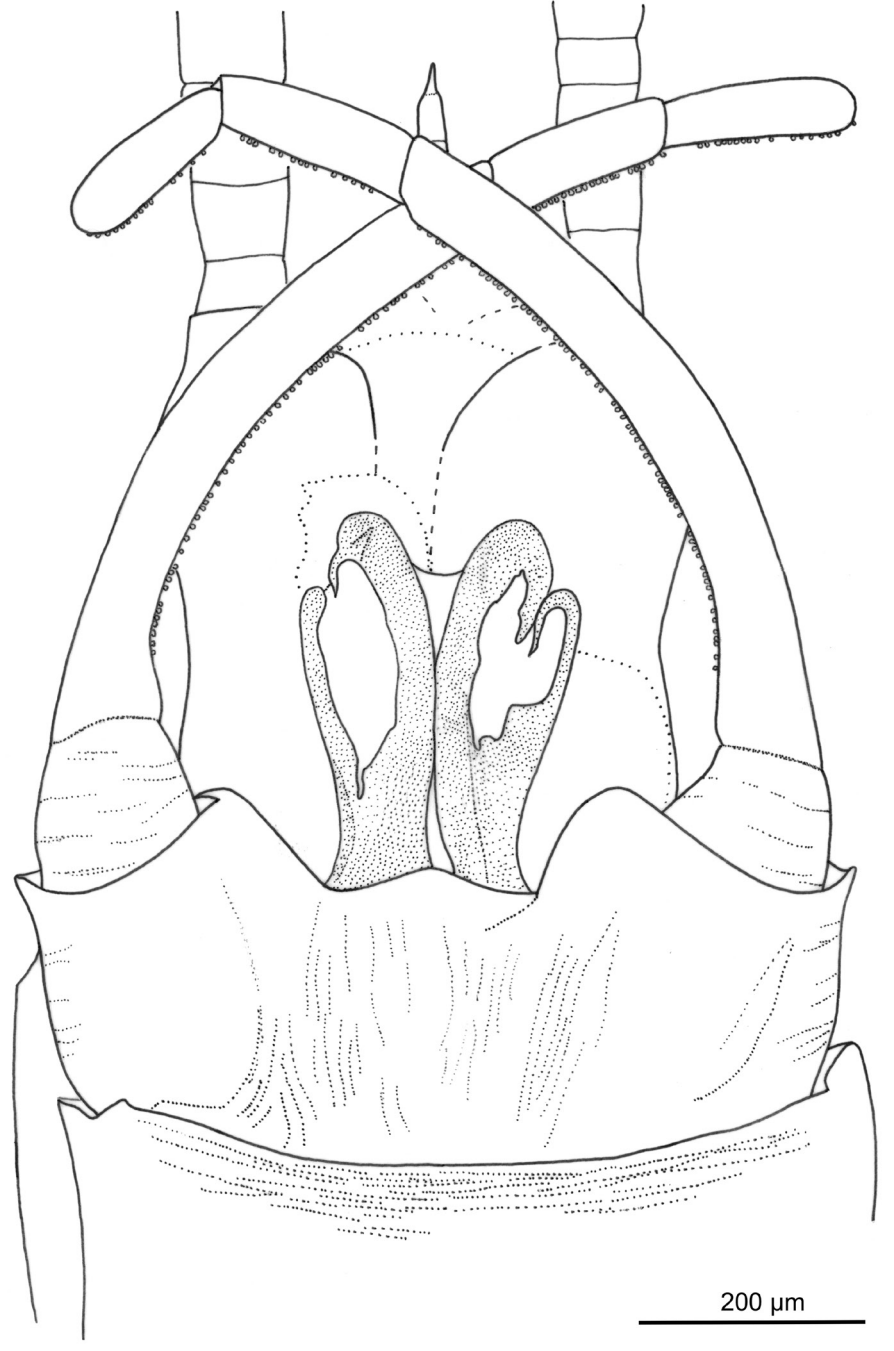
*Siphloplecton landolti* sp. nov., SMNS, BB–2377, holotype, male imago (line drawing): genitalia in ventral view.

Figures 5 , 6 , 7 ; Table 1

###### Material examined.

***Holotype*.** Male imago in Baltic amber (Eocene), SMNS BB–2377. Well preserved specimen in clear amber, well visible in dorsoventral aspect. Each of both forewings bent upwards approximately at half length (left forewing partly twisted); left hind wing twisted; genitalia visible in ventral and, partly, lateral view.

For measurements see Table 1.

###### Description.

Colouration pale, dorsal side slightly darker than ventral side. Brownish diffuse maculation on thorax and abdomen preserved. Ocelli well preserved. Eyes large, medially contiguous; brownish maculation on eye surface partly preserved; antennae longer than head (Figs 5A–C, E, 6A–B).

***Thorax*** with traces of brownish pigmentation on dorsal and ventral sides; “Verlumung” mainly on lateral sides near wing bases. Lateroparapsidal suture elongate, typical for *Siphloplecton*; no conspecific pigmentation around lateroparapsidal suture; mesonotal suture medially bulged (Fig. 6A); furcasternal protuberances of mesothorax contiguous (Fig. 6B); lateral aspect of thorax hardly visible.

***Wings*** translucent, hyaline, not pigmented, venation well visible; pterostigma translucent, without pigmentation, with simple veins. Cubital field of forewing well visible on right wing only (twisted on left wing): one distinct pair of intercalary veins (iCu2 and iCu3) situated close to CuP and connected with CuP and CuA; one long intercalary vein (iCu1) connected with CuA and also with iCu2 by crossveins; additional short intercalaries present, ending at hind margin of wing (one of them between iCu1 and iCu2, basally joining both veins) (Fig. 5C). Hind wings with triads RS, MA and MP, 0.31× forewing length; costal process bluntly pointed (Fig. 5D).

***Legs*** damaged; both right fore- and hind legs lost; tarsus of left foreleg only partly preserved (for details see Table 1). Tibia of left foreleg paler than femur and tarsus. Left foretibia with strong, sharply pointed setae along outer margin. Tibia of left middle and hind legs black; preserved part of first tarsomere of left middle leg black. Tibia of middle and hind legs with trace of tibiopatellar suture; first tarsomere of middle and hind legs fused with tibia. Tarsal claws lost (Fig. 5A–B).

***Abdominal*** segments completely preserved, sterna visibly paler than terga; genitalia dark.

***Styliger*** plate angulate, mediocaudally deeply incised; these incisions with pronounced, broad, triangular projections; medial projection not large, blunt apically (Figs 5F, 7). Basal segment of forceps basally relatively wide; forceps 4-segmented, segment 4 approximately 3.30 times longer than wide; length ratio of segment 3 to segment 4 approximately 1:1. Penis lobes distinctly elongated, well separated from each other apically, ellipsoidal; medial sclerite rounded apically; lateral and medial sclerite markedly separated on outer side with inconspicuous incision between sclerites; features of ventral surface of penis lobes not visible (Figs 5F, 7).

***Paracercus*** vestigial; cerci partly lost.

###### Comments.

The new species can be placed within the *jaegeri* species group based on the characteristic shape of the penis lobes, the arrangement of cubital intercalaries of the forewing and the sharply pointed setae along the outer margin of the foretibia. *Siphloplecton landolti* sp. nov. can be separated from the closely related *S. jaegeri* by (1) shape of styliger with relatively small and apically rounded medial projection; (2) relatively wide base of basal forceps segment compared to adjoining apical part of plate; (3) proportions of last forceps segments; (4) ellipsoidal shape of penis lobes with medial sclerite rounded at tip.

The body measurements of *S. landolti* sp. nov. are comparable to other representatives of the *S. jaegeri* species group, but the ratio of hind/forewing length is lower in comparison with *S. jaegeri* itself.

###### Etymology.

Following our tradition of naming new fossil species of *Siphloplecton* after Swiss ephemeropterists, this species is named after Peter Landolt, Lausanne, to honour his contributions to the knowledge of Swiss mayflies.

##### Siphloplecton studemannae sp. nov.

Taxon classificationAnimaliaEphemeropteraMetretopodidae

EC67D71F-662D-5B5D-BF37-B9A705B11950

http://zoobank.org/2D1C8631-DAFF-4B84-B10D-7D7FAB3E5334

**Figure 8.**
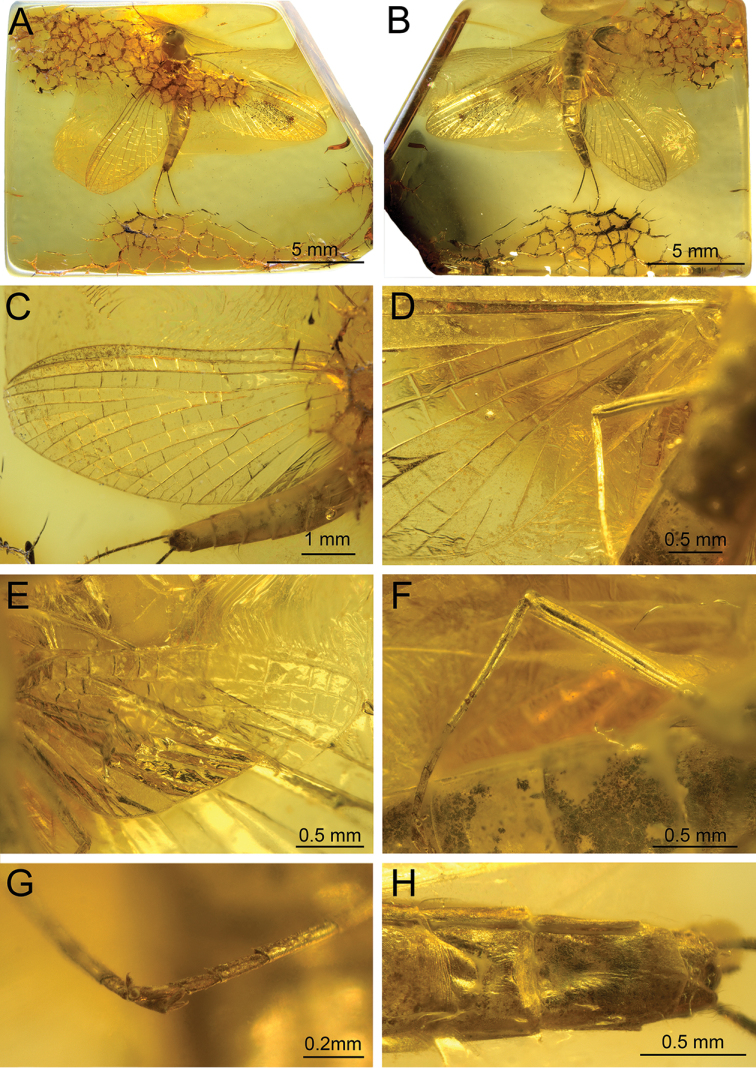
*Siphloplecton studemannae* sp. nov., SMNS, BB–2626, holotype, female imago (photographs) **A** general dorsal view **B** general ventral view **C** left forewing in dorsal view **D** wing base and cubital field of right forewing in ventral view **E** left hind wing in ventral view **F** right hind leg in medial view **G** claws of hind legs **H** abdominal segments VII–X in ventral view.

**Figure 9.**
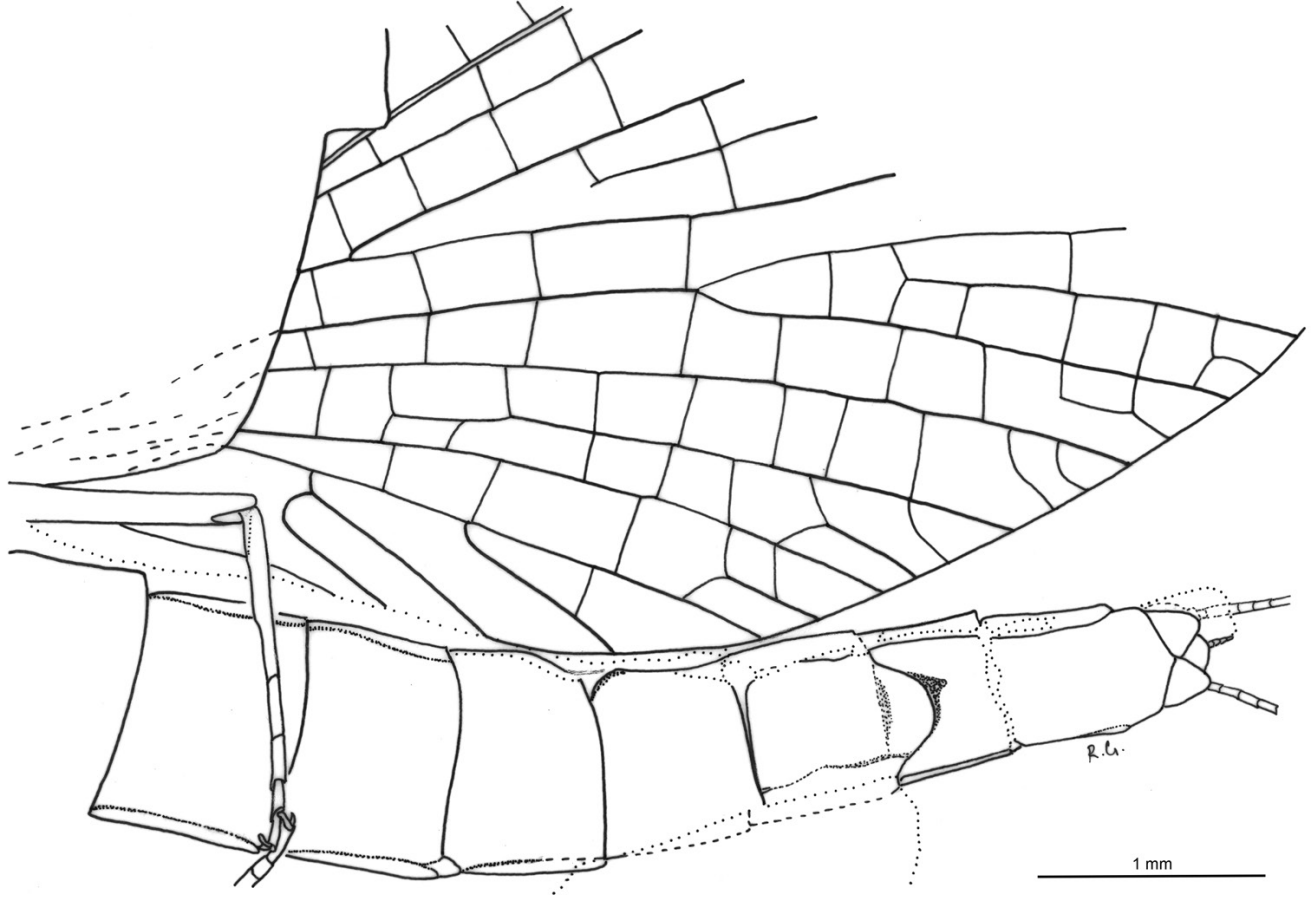
*Siphloplecton studemannae* sp. nov., SMNS, BB–2626, holotype, female imago (line drawing): abdomen and part of left forewing in ventral view.

**Figure 10.**
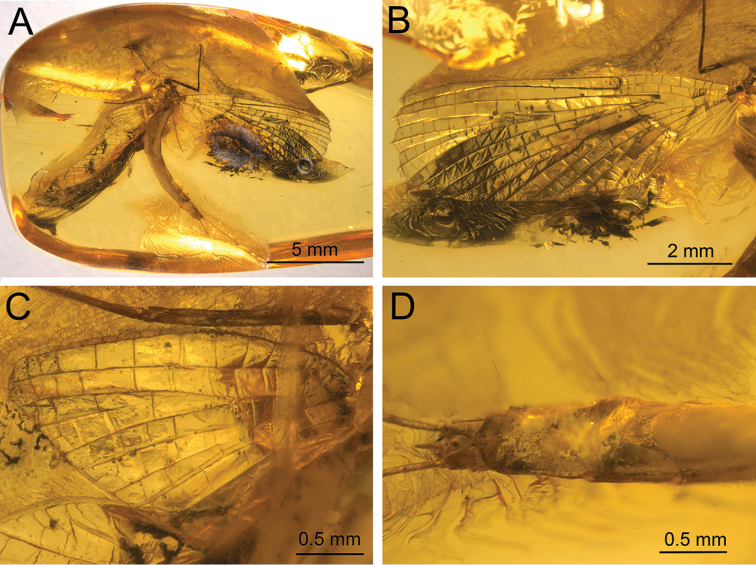
*Siphloplecton studemannae* sp. nov., MNHK, MP/1626, paratype, female imago (photographs) **A** general ventral view **B** left forewing in dorsal view **C** right hind wing in ventral view **D** abdominal segments VII–X in ventral view.

**Figure 11.**
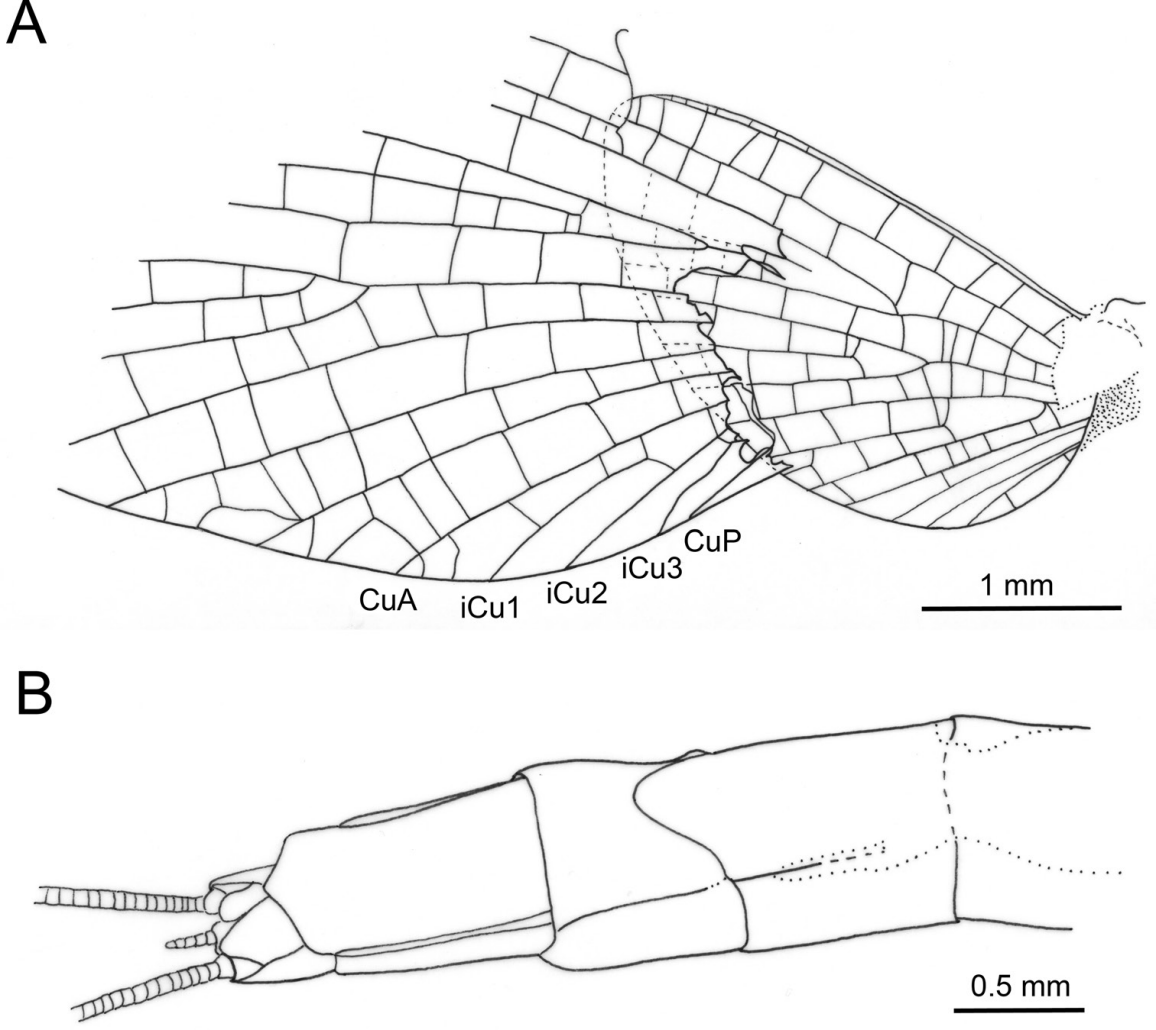
*Siphloplecton studemannae* sp. nov., MNHK, MP/1626, paratype, female imago (line drawings) **A** fragments of left fore- and hind wings, dorsal view **B** abdominal segments VII–X in ventrolateral view.

Figures 8 , 9 , 10 , 11 ; Table 1

###### Material examined.

***Holotype*.** Female imago in Baltic amber (Eocene), SMNS BB–2626. Well preserved specimen in clear amber, well visible in dorsoventral aspect. Right foreleg lost; hind wings (especially left wing) twisted. Head and thorax covered by “Verlumung”. Additionally, piece of amber with numerous cracks, thus details of thoracic terga invisible, and thorax only partly visible from ventral side. For measurements see Table 1.

***Paratype*.** Female imago in Baltic amber (Eocene), MNHK, MP/1626. Partly damaged specimen, visible in ventral aspect. View on body hampered by resin influxes, numerous cracks, and considerable “Verlumung”, so thoracic sutures are hardly visible. Head lost. Pronotum damaged, its structure invisible. Distal part of mesonotum, metanotum and abdominal segments I−IV dorsally covered by plant tissue. Right forewing twisted along its length; left forewing lacks its basal part and distal end. Right foreleg, right middle leg, left middle tarsus, and both hind legs lost. Cerci only partly preserved. For measurements see Table 1.

###### Description of holotype.

General colour of body pale, yellow to yellowish-brown. Ventral side of body slightly darker than dorsal side. Ocelli well preserved. Eyes medially separated, but approximated (Fig. 8A). Distance between eyes 0.22× of head width. Antennae complete, slightly longer than head, pale.

***Thorax*** ventrally yellowish coloured by “Verlumung”, mainly in posterior part. Furcasternal protuberances of mesothorax contiguous (Fig. 8B); lateral aspect of thorax not visible.

***Wings*** translucent, hyaline, not pigmented. Wing venation well visible only from ventral side; basal part of wings covered by numerous cracks from dorsal side. Cubital field of both forewings with one pair of intercalary veins (iCu2, iCu3) towards CuP and one additional vein (iCu1) near CuA (Figs 8C–D, 9). Right hind wing hardly visible, twisted; left hind wing completely preserved with three pairs of triads, 0.34× of right forewing length. Costal process bluntly pointed apically and small (Fig. 8E).

***Legs*** relatively well preserved, except forelegs (right is lost; left incomplete). Measurements of leg segments in Table 1. Tibiae of middle and hind legs each with trace of tibiopatellar suture; first tarsomere of middle and hind legs fused with tibia (Fig. 8F). Tarsi with 5 tarsomeres; tarsal claws dissimilar: one hooked and one blunt (Fig. 8G).

***Abdominal*** segments completely preserved, pale; terga VIII–X slightly darker than remaining terga. Abdominal sterna slightly covered by “Verlumung”. Subgenital plate relatively broad, 2.00× as wide as long, convex and rounded apically. Subanal plate not elongated, narrow, with pointed tip (Figs 8H, 9). Paracercus poorly visible, vestigial, with at least 4 visible segments; cerci dark, partly preserved.

###### Description of paratype.

Body colouration light, yellowish-brown to brown. Irregular brown, dirty brown to black maculation over body, especially on forewings and legs (Fig. 10A).

Details of mesonotum hardly visible; mesonotal suture typical for *Siphloplecton*, original lateroparapsidal suture colouration invisible due to resin influx. Furcasternal protuberances contiguous, distinctly brown; other parts of mesosternum paler.

***Pterostigma*** with anastomosed crossveins. Cubital field of right forewing well preserved; iCu1 basally connected to CuA and iCu2 by crossveins, 2 additional CuA-iCu1 crossveins present. Pair of intercalaries iCu2 and iCu3 basally connected to each other; further crossveins connect to CuA and CuP (Figs 10B, 11A). Hind wings relatively long, approximately 0.40× of length of preserved right forewing; costal processes hardly visible. Hind wings with triads RS, MA and MP; costal process (visible only on right hind wing) small and blunt (Fig. 10C).

***Left*** foreleg intensely brown to blackish distally; middle legs paler, yellow to yellowish-brown. Preserved foreclaw dissimilar (one claw hooked, one claw blunt).

Most of ***abdominal*** segments ventrally covered with “Verlumung”. Preserved sterna paler than terga. Subgenital plate not elongate, approximately 1.80× wide as long, widely rounded apically. Subanal plate not elongate, pointed at tip (Figs 10D, 11B). Paracercus 5-segmented.

###### Comments.

We allocate *Siphloplecton studemannae* sp. nov. within the *S. jaegeri* species group based on the presence of three distinctive intercalaries (grouped in one pair and one additional intercalary vein) in the cubital field of both forewings. The same arrangement of intercalary veins was described and figured for the holotype of *S. jaegeri* by Staniczek and Godunko (2012: 73, 74, fig. 10b). Other diagnostic characters of this species group, namely the presence of sharply pointed setae at the outer margin of the foretibia, cannot be observed in the type specimens of *S. studemannae* sp. nov. So it cannot be excluded that this character is present in the females of this fossil species, similarly to the different occurrence of this character in the two sexes of extant species (Berner 1978).

The new species can be characterized by the presence of the following characters: (1) eyes separated, but close-set; (2) cubital field of forewings with one pair of intercalaries and one intercalary vein connected with CuA; (3) subgenital plate relatively broad with width/length ratio 1.80−2.00. This combination of characters definitely separates the described females of *S. studemannae* sp. nov. from all other previously known Recent and fossil taxa.

*Siphloplecton studemannae* sp. nov. is currently the only species of the genus *Siphloplecton* described from female specimens that can also be attributed to a certain species group. This is only possible due to the unique arrangement of cubital intercalaries, which is characteristic for the *S. jaegeri* species group. The previously described *S. barabani* and *S. hageni*, also only known from female specimens, do not provide any clear distinguishing characters that would allow their placement in one of the other fossil species groups defined for *Siphloplecton*, which can only be grouped based on similarities in the male genitalia.

###### Etymology.

Following our tradition of naming new fossil species of *Siphloplecton* after Swiss ephemeropterists, this species is named after Denise Studemann, Lausanne, to honour her contributions to the knowledge of Swiss mayflies.

##### *Siphloplecton* spp. (*jaegeri* species group)

###### Siphloplecton

Taxon classificationAnimaliaEphemeropteraMetretopodidae

sp. 5

B80DCBA3-7FCD-52A9-A005-BA5913ADDA0E

**Figure 12.**
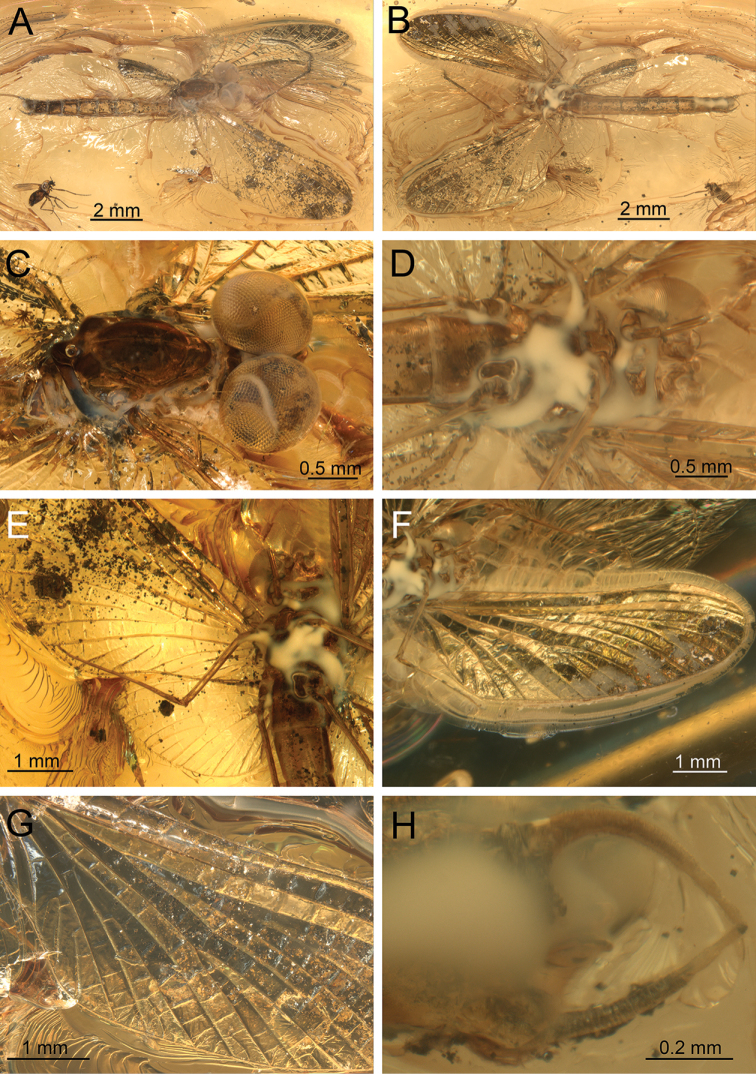
*Siphloplecton* sp. 5, CCHH, BaB 1159/5, male imago (photographs) **A** general dorsal view **B** general ventral view **C** head and thorax in dorsal view **D** head and thorax in ventral view **E** base of right forewing and hind wing in ventral view **F** left forewing in ventral view **G** cubital field of left forewing in ventral view **H** genitalia in ventral view.

**Figure 13.**
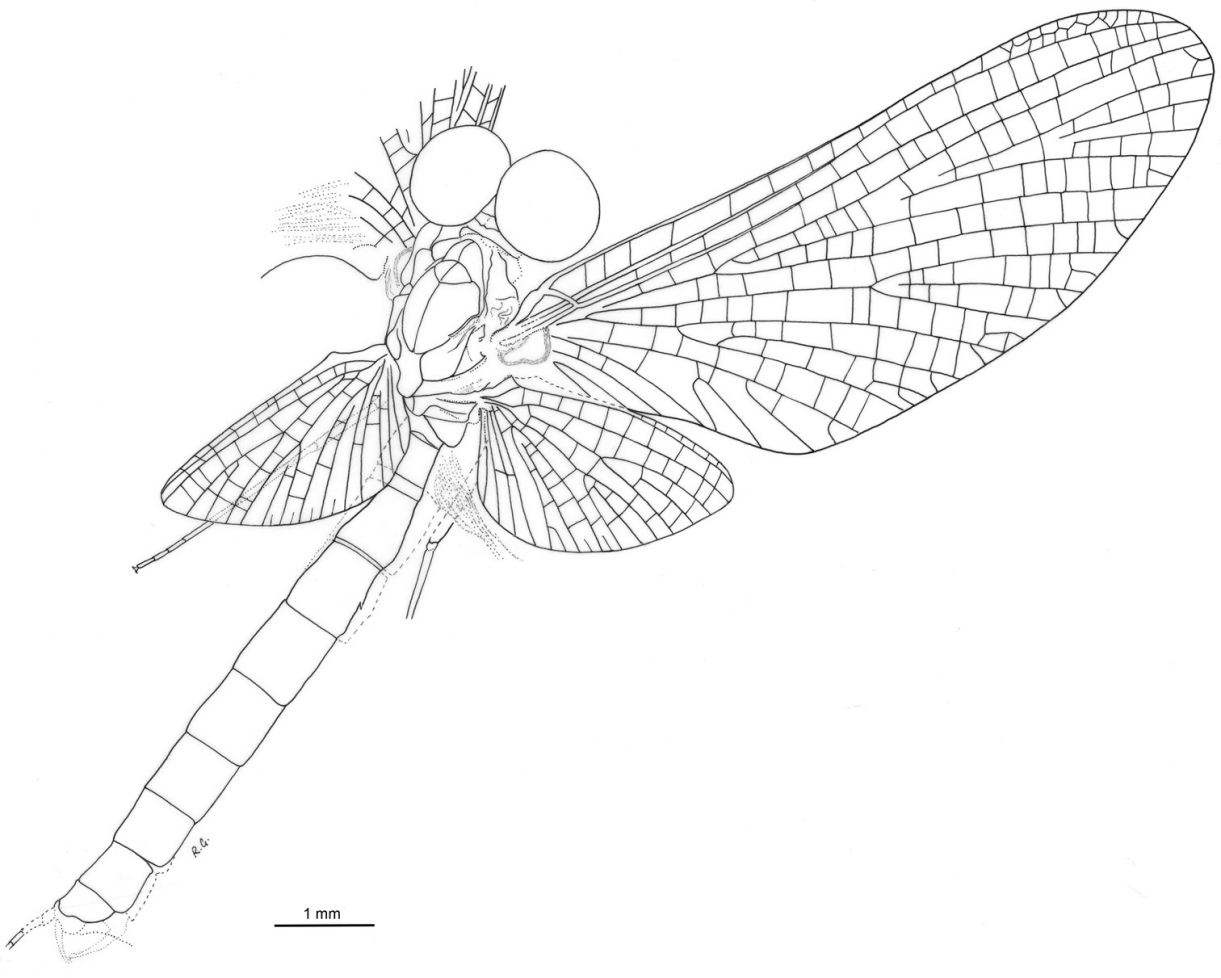
*Siphloplecton* sp. 5, CCHH, BaB 1159/5, male imago (line drawing): general dorsal view of body, right forewing, left and right hind wings.

Figures 12 , 13 ; Table 1

####### Material examined.

Male imago in Baltic amber (Eocene), CCHH, BaB Nr. 1159/5. A generally completely preserved specimen, well visible in dorsoventral aspect in translucent amber (Fig. 12A–B). Some resin influxes around specimen. Right foreleg and right cercus lost. Dorsal side of body only with several very small spots of “Verlumung”. Ventral side of head, thorax and, partly, abdominal sterna intensively covered with “Verlumung”. For this reason, some aspects of mesosternum and the shape of the genitalia are poorly visible. One female nonbiting midge (Diptera: Chironomidae) is embedded in the same stone.

Relatively pale specimen, yellowish to brown. Wings with artificial, irregular dark spots; distal portions of forewings and right hind wing with distinct concentration of such spots (Fig. 12E). Legs uniformly coloured. For measurements see Table 1.

####### Description.

***Head*** light brown. Eyes large, slightly flattened, pale, dirty yellowish. Several brownish spots on eye surface. Ocelli and antennae of same colouration as head, completely preserved; antennae slightly longer than head (Fig. 12C, E).

***Thorax*** with traces of brownish pigmentation on dorsal and ventral sides, brown. Pronotum well preserved. Arrangement of thoracic sutures on dorsal and ventral sides of mesothorax typical for *Siphloplecton* (Figs 12C–D, 13). Most of head and thorax ventrally covered with “Verlumung”. Lateral aspect of thorax hardly visible.

***Wings*** basally translucent, hyaline, with well visible longitudinal and transversal venation. Pterostigmatic area hyaline, opaque, with several anastomosed veins. Cubital field of forewings with well visible pair of intercalary veins connected with CuA, and one additional, well developed vein also directed toward CuA (Figs 12E–G, 13). Hind wings with triads RS, MA and MP, 0.36× forewing length; costal process bluntly pointed and small (Figs 12E, 13).

***Legs*** yellowish to light brown. Several pointed spines at outer margin of foretibia. Characteristics of legs similar to those of other representatives of *Siphloplecton*. Measurements of leg segments in Table 1.

***Abdominal*** segments completely preserved; abdominal terga slightly paler than sterna.

***Styliger plate*** only partly visible, most of styliger base covered with “Verlumung”. Thus, shape of styliger projections not clearly visible (Fig. 12H). Basal segment of forceps basally not widened, distinctly narrower than adjoining apical part of plate (character well visible only in right clasper); forceps 4-segmented, segment 4 approximately 3.20 times longer than wide; length ratio of segment 3 to segment 4 approximately 1:1 (Fig. 12H).

***Penis*** lobes covered with “Verlumung” (especially left lobe); penis lobes elongated, relatively narrow and separated from each other apically; medial sclerite rounded apically; lateral and medial sclerites probably separated on outer side (poorly visible) (Fig. 12H).

***Paracercus vestigial***, at least 3-segmented; left cercus slightly damaged; right cercus lost.

####### Comments.

This species is closely related to *S. landolti* sp. nov. due to the similar proportions of the forceps segments and shape of the penis lobes, especially the apical portion of the medial sclerite, and the presence of 2+1 intercalary veins in the cubital field of the forewings. This last character also confirms the inclusion of *Siphloplecton* sp. 5 within the *S. jaegeri* species group. At the same time, we could not confirm its conspecificy with *Siphloplecton landolti* sp. nov. or other species, since the details of the male genitalia are poorly visible.

###### Siphloplecton

Taxon classificationAnimaliaEphemeropteraMetretopodidae

sp. 6

2FF6F668-DE38-553F-973B-7B0C4CDC0DA3

Figure 14 ; Table 1

####### Material examined.

Male subimago in Baltic amber (Eocene), MNB, MB.I 7372, specimen originally labeled as: “6. Pseudoneuroptera III Ephemeridae”; “Museum für Naturkunde Berlin”; “Paläontologisches Museum”; “Slg.: Künow Inv. Nr.: Nr. 268–294 nur noch 9 Stück vorgefunden”; “Ephemeriden“; “Siphloplecton cf. jaegeri subim. male Nr.: 272”.

Completely preserved specimen, well visible in lateral aspect. Details of head hardly visible due to resin influxes and small cracks in stone. Left forewing arcuated at half length; left hind wing twisted. Terga IX–X and, partly, genitalia covered by “Verlumung”. For measurements see Table 1.

####### Description.

General colouration of body brown to dark brown. Head brown with paler antennae; eyes large, medially contiguous, uniformly brown coloured.

***Thorax*** intensely brown; details of terga hardly visible; mesonotal suture typical for *Siphloplecton*; lateroparapsidal suture not visible. Thorax ventrally hardly visible, but furcasternal protuberances of mesothorax contiguous.

**Figure 14.**
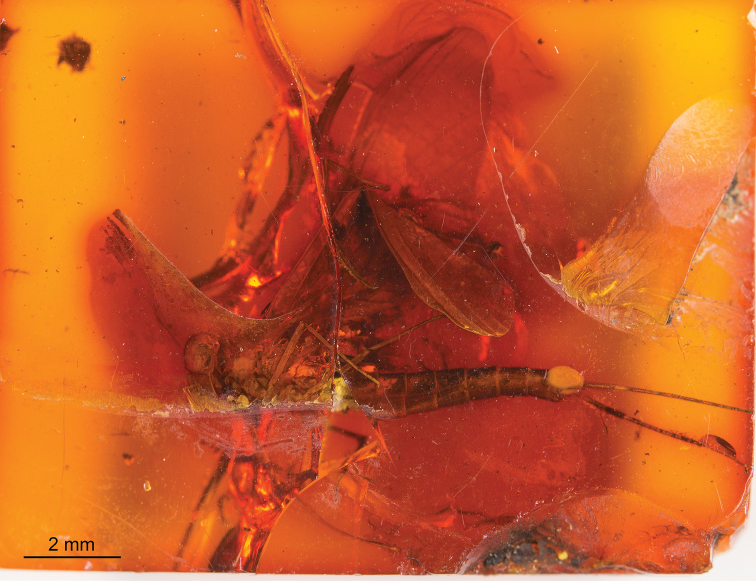
*Siphloplecton* sp. 6, MNB, MB.I 7372, male subimago (photograph): general lateral view, left side.

***Wings*** opaque. Forewings with several irregular dark spots (probably an artefact of fossilisation). Pterostigmatic area with 7–8 anastomosed veins. In cubital field of forewing one pair of intercalaries close to CuP (connected with CuP and CuA); one additional intercalary vein close to CuA. Hind wings with triads RS, MA and MP, 0.32× forewing length; costal process bluntly pointed (Fig. 14).

***Legs*** typical for the genus *Siphloplecton*.

***Abdominal*** segments completely preserved; terga darker than sterna.

Shape of ***plate*** poorly visible in ventral view, but generally close to the one of *S. landolti* (deeply incised mediocaudally, angulate, with relatively small medial projection). Shape of penis lobes hardly visible because of “Verlumung”. Visible part of penis lobes with apical sclerites rounded at tip.

***Cerci*** partly damaged; paracercus vestigial.

####### Comments.

Due to the general shape of the styliger and penis lobes, and proportions of the fore/hind wings, this specimen belongs with high probability to *S. landolti* sp. nov. Some of the observed differences might be related to differences between subimago and imago, or may be due to the bad preservation of the genitalia. However, in order to avoid any taxonomic confusion, we refrain from attributing *Siphloplecton* sp. 6 to a certain species within *Siphloplecton*.

## Discussion

The first classification of extant representatives of the genus *Siphloplecton* into species groups was undertaken by Berner (1978) in his work dedicated to the taxonomy of Metretopodidae. That grouping was based on wing pigmentation, the shape of the setae along the outer margin of the foretibia, and penis shape (Berner 1978: 96; Staniczek and Godunko 2012: 59–61, figs 1–2). Staniczek and Godunko (2012), for the first time, added fossil *Siphloplecton* species from Eocene Baltic amber to Recent species groups. In this contribution, *S. picteti* and *S. demoulini* were attributed to the *S. basale* species group established by Berner (1978). At the same time, the remaining investigated taxa, *S. barabani* and *S. hageni*, were neither attributed to the *basale* species group nor to the *interlineatum* species group. This was due to these species having only been described from female imagines, and no traces of wing pigmentation were preserved. *Siphloplecton jaegeri* was also excluded from previously known species groups for peculiarities in the genitalia and for the presence of pointed setae along the outer margin of the foretibia (Staniczek and Godunko 2012). In fact, these characters pointed to a relatively isolated position of *S. jaegeri* within both fossil and recent species of *Siphloplecton*.

Based on the investigation of further male specimens, Staniczek and Godunko (2016) established another two *Siphloplecton* species groups for four fossil species: *S. picteti* and *S. sartorii* were attributed to the *S. picteti* species group, and *S. demoulini* and *S. gattolliati* to the *S. demoulini* species group. At the same time, due to the poor preservation of the re-discovered lectotype of *S. macrops*, Staniczek and Godunko (2016) concluded that it is neither possible to define any distinguishing characters for this species nor to attribute it to any of the species groups proposed earlier. They also refrained from placing female specimens in the *S. sartorii* or *demoulini* species groups.

Representatives of the genus *Siphloplecton* are relatively abundant components of the mayfly fauna from Eocene Baltic amber. Judging from the museum and private collections investigated by us, this genus is not less abundant than *Paraleptophlebia* Lestage, 1917 (Leptophlebiidae), which is another commonly found mayfly genus in Baltic amber. Taxonomically, the genus *Siphloplecton*, with ten fossil and nine contemporary Nearctic species, is rather diverse.

### Key to males of *Siphloplecton* from Eocene Baltic amber

**Table:** 

1	Four long intercalary veins grouped into two pairs in the cubital field of the forewings between CuA and CuP (Staniczek and Godunko 2012: 66, 76, figs 4C, 12C; Staniczek and Godunko 2016: 14, 19, 23, figs 7A, 11A, 13A)	**2**
–	Three long intercalary veins grouped into one pair and one separate intercalary vein in the cubital field of the forewings between CuA and CuP (Figs 1D, 2, 4A, 8D, 9, 10B, 11A, 12E, G, 13) [*S. jaegeri* species group]	**5**
2	Outer margin of foretibia with two or more stout, pointed setae; penis relatively short, its tip barely reaching distal margin of basal forceps segment [*S. picteti* species group]	**3**
–	Outer margin of foretibia without stout, pointed setae; penis elongated, its tip significantly projecting beyond plate, reaching one fourth of forceps segment 2 [*S. demoulini* species group]	**4**
3	Basal segment of forceps nearly square; segment 4 of forceps moderately elongated (segment length/width ratio 2.58−2.72); forceps segments 3 and 4 of about same length; penis lobes contiguous almost along entire length, separated only by a shallow, broad, U-shaped cleavage apically; lateral sclerites of penis lobes relatively slender, only slightly expanding laterally (Staniczek and Godunko 2012: 64, fig. 3b; Staniczek and Godunko 2016: 12, 14–16, figs 6D, 7B, 8E, 9B)	***S. picteti***
–	Basal segment of forceps slim and elongated; segment 4 of forceps clearly elongated (segment length/width ratio 3.38); forceps segment 3 slightly longer than segment 4; penis lobes well separated apically by a wide, V-shaped cleft; lateral sclerites of penis lobes distinctly broad and prominent laterally (Staniczek and Godunko 2016: 18, 19, figs 10H, 11B)	***S. sartorii***
4	Median projection of plate slightly prominent, triangular (Staniczek and Godunko 2012: 76, 77, figs 12d, 13c)	***S. demoulini***
–	Median projection of plate absent (Staniczek and Godunko 2016: 22, 23, figs 12F, 13B)	***S. gattolliati***
5	Median projection of plate well protruded, broad, triangular-shaped; basal segment of forceps basally markedly narrower than adjoining apical part of plate; penis lobes distinctly elongated, nearly triangular-shaped apically (Figs 1E, 3D, 4B; Demoulin 1968: 253, fig. 18b; Staniczek and Godunko 2012: 74, fig. 11c)	***S. jaegeri***
–	Median projection of plate relatively small, rounded apically; basal segment of forceps basally relatively wide in comparison to adjoining apical part of plate; penis lobes distinctly elongated, smoothly rounded apically (Figs 5F, 7)	***S. landolti***

## Supplementary Material

XML Treatment for Siphloplecton jaegeri

XML Treatment for Siphloplecton jaegeri

XML Treatment for Siphloplecton landolti

XML Treatment for Siphloplecton studemannae

XML Treatment for Siphloplecton

XML Treatment for Siphloplecton
